# SMOTE-Based Automated PCOS Prediction Using Lightweight Deep Learning Models

**DOI:** 10.3390/diagnostics14192225

**Published:** 2024-10-05

**Authors:** Rumman Ahmad, Lamees A. Maghrabi, Ishfaq Ahmad Khaja, Louai A. Maghrabi, Musheer Ahmad

**Affiliations:** 1 Department of Computer Engineering, Jamia Millia Islamia, New Delhi 110025, India; rummanahmad05@gmail.com (R.A.); ishfaqkhawaja312@gmail.com (I.A.K.); 2 Department of Endocrinology and Metabolism, Internal Medicine, Dr. Soliman Fakeeh Hospital, Jeddah 23323, Saudi Arabia; lamaghrabi@fakeeh.care; 3 Department of Software Engineering, College of Engineering, University of Business and Technology, Jeddah 23411, Saudi Arabia; l.maghrabi@ubt.edu.sa

**Keywords:** polycystic ovary syndrome (PCOS), deep learning, 1D CNN, LSTM, SMOTE

## Abstract

Background: The reproductive age of women is particularly vulnerable to the effects of polycystic ovarian syndrome (PCOS). High levels of testosterone and other male hormones are frequent contributors to PCOS. It is believed that miscarriages and ovulation problems are majorly caused by PCOS. A recent study found that 31.3% of Asian women have been afflicted with PCOS. Healing women with life-threatening disorders associated with PCOS requires more research. In prior research, methods have involved autonomously classified PCOS using a number of different machine learning techniques. ML-based approaches involve hand-crafted feature extraction and suffer from low performance issues, which cannot be ignored for the accurate prediction and identification of PCOS. Objective: Hence, predicting PCOS using cutting-edge deep learning methods for automated feature engineering with better performance is the prime focus of this study. Methods: The proposed method suggests three lightweight (LSTM-based, CNN-based, and CNN-LSTM-based) deep learning models, incorporating SMOTE for dataset balancing to obtain a valid performance. Results: The proposed three models tend to offer an accuracy of 92.04%, 96.59%, and 94.31%, an ROC-AUC of 92.0%, 96.6%, and 94.3%, the number of parameters of 6689, 297, and 13285, and a training time of 67.27 s, 10.02 s, and 18.51 s, respectively. In addition, the DeLong test is also performed to compare AUCs to assess the statistical significance of all three models. Among all three models, the SMOTE + CNN models performs better in terms of accuracy, precision, recall, AUC, number of parameters, training time, DeLong’s *p*-value over the other. Conclusions: Moreover, a performance comparison is also carried out with other state-of-the-art PCOS detection studies and methods, which validates the better performance of the proposed model. Thus, the proposed model provides the greatest performance, which can lead to a reduction in the number of failed pregnancies and help in finding PCOS in the early stages.

## 1. Introduction

For AI applications on resource-constrained devices like mobile phones, where processing power and memory capacity are limited, the model needs to be lightweight. A study by Azamossadat Hosseini [1] developed a mobile application utilizing lightweight CNN models, including EfficientNetB0, MobileNetV2, and NASNet Mobile, to distinguish B-ALL (B-cell acute lymphoblastic leukemia) from healthy cells. By tailoring the architecture for mobile environments, the study achieved 100% sensitivity and specificity, showcasing the effectiveness of lightweight models for real-time, accessible medical diagnosis while reducing the reliance on expensive, invasive procedures. Polycystic ovary syndrome (PCOS), also acknowledged as hyperandrogenic syndrome, is a hormonal disruption affecting a significant proportion of women who are in the period of reproduction. In addition to causing irregular or delayed menstrual periods, PCOS is related to an increased amount of male hormones. The causes of polycystic ovary syndrome (PCOS) have not been identified. However, early detection and treatment, as well as a reduction in body weight, may lessen the likelihood of developing long-term issues. According to [2], around 10% of all females in the age range when they can have children are affected by this condition.

An imbalance of sex hormones is the root cause of PCOS. Cysts form in the ovary of females when there is an increase in the number of androgens. These tumors grow over time, eventually becoming large enough to hamper the ovulation process. A woman with PCOS, because of the disturbance of fertilization it causes, has a reduced probability of becoming pregnant [3]. A sleep disorder, irritable and angry behavior, acne, oily skin, weight gain, headaches, and male-like hair growth on the back, stomach, face and chest are also common symptoms of PCOS [4]. Even though PCOS is commonly thought of as a lifestyle disorder, the specific causes for its emergence are not yet fully understood. The repercussions of PCOS may be minimized with appropriate physical activity, a nutritious diet, and the maintenance of body weight. But it is difficult to entirely solve this disease. The majority of women do not discover that they have PCOS until they take a test to determine whether or not they are pregnant [5]. While cysts can be identified using ultrasounds, and androgen concentration can be evaluated using medical tests, there is no accurate and relevant test to identify PCOS [6,7]. An early diagnosis of PCOS symptoms assists in making essential healthy decisions. Women with such a disorder have a high risk of miscarriage. Also, gynecological carcinoma occurs in women with PCOS due to infertility [8]. Hence, it is vital to detect PCOS at an early stage to reduce miscarriages. A recent study found that PCOS is detected in 31.3% of women in Asia, 4.8% of women of White American heritage, 8% of women of Black American ancestry, 6.8% of women in Spain, and 4.8% of women in America [9]. Regular exercise for women lowers the concentration of androgen and biochemical hyperandrogenism [10,11,12,13,14]. Research indicates that with an upsurge in age, PCOS symptoms become less severe and menopause occurs [15,16,17].

Mobile apps have become vital for human progress, including pandemic control. This study reviews how mobile applications were used to diagnose COVID-19 during the pandemic. Out of 535 studies, 42 were selected, focusing on AI-based diagnosis, contact tracing, data collection, and visualization. AI techniques, specifically deep learning models like convolutional neural networks, proved effective in identifying COVID-19 cases using symptoms, cough sounds, and radiological images. The study highlights the potential of mobile apps, integrated with technologies like IOT and cloud computing, to enhance rapid diagnosis and improve disease management in future pandemics [18].

Traditionally, doctors rely on ultrasound imaging to identify affected ovaries, but distinguishing between benign, PCOS-related, or cancerous cysts can be challenging. This study utilizes a deep learning model for classifying PCOS from ultrasound images. A trained dataset is used for feature extraction and accuracy measurement. The paper also discusses methods for reducing noise, segmenting regions of interest, and improving cyst detection accuracy to support early diagnosis and treatment [19]. In the approaching years, disease diagnosis and treatment are likely to be significantly impacted by AI and its subfields, which are steadily becoming popular in daily life [20]. This work uses AI to diagnose PCOS in an unexplored domain. Traditional diagnosis is complicated and time-consuming due to the complexity of all the factors in a woman’s life that might lead to PCOS and the trouble in evaluating sonographic cyst visualization. Hence, PCOS diagnosis may very well be improved by our proposed computational technique, which will also benefit millions of women. A list of abbreviations used in this paper is presented in Table 1.

The remaining portion of our research study is as follows: An associated review of the literature on PCOS is included in Section 2 of this paper. In Section 3, the preliminary details of the CNN, LSTM, and SMOTE are presented. The proposed methodology is discussed in Section 4. A performance evaluation of the proposed models designed in this paper for PCOS is conducted in Section 5. Lastly, we conclude our study in Section 6.

## 2. Related Works

This section discusses an analysis of the relevant prior research studies and frequently utilized cutting-edge research on PCOS prediction. These papers were chosen because of their relevance to the topic at hand.

PCOS disorder is among the most prevalent health issues diagnosed in younger women with a variety of medical symptoms [21]. The early identification of PCOS is very much needed to avoid long-term issues. To accomplish this objective, experts used a wide variety of machine learning strategies [22,23]. The random forest method provided an accuracy score of 96% in PCOS diagnosis as suggested in [24]. Based on the optimized chi-squared method, a unique machine learning-based strategy, CS-PCOS, was proposed to select the most prominent feature from thale dataset for the detection of PCOS in [25]. Reference [26] presented an online application that allows women to quickly and simply assess their risk of developing the disorder from the convenience of their residences while they wait for assistance to become available. The authors in [27] tried a variety of machine learning algorithms, out of which the KNN classifier achieved the best results. Along with machine learning-based methods investigated in [28,29,30], the authors utilized feature selection techniques to lessen the subset of features and focus on the most relevant ones. Different algorithms such as random forest, multi-layer perceptron, naïve Bayes, etc., provide great and effective results for these refined features. Using microarray and RNA-seq datasets, a new diagnostic framework was developed in [31] for PCOS by employing random forest and artificial neural network techniques, which exhibited a superior generalization ability in microarray data. Similarly, a method was also presented in [32], which used the feature selection technique along with random forest as a predictive model.

A study was conducted using Raman spectroscopy in [33] by collecting profiles of blood serum from women with PCOS as well as women without it. The blood profiles that were obtained revealed clear differences in brightness that were taken from people with PCOS in a comparison with a person without the condition. According to the observations, the ratio of lipids to proteins has the potential to function as a useful PCOS indicator that may be identified using Raman spectroscopy. Apart from machine learning-based methods, researchers are also utilizing deep learning strategies by making use of CNNs to extract relevant features from ultrasound image datasets. Reference [34] also proposed a deep learning model for the prediction of PCOS using ultrasound images. The authors of [35] developed a count-based ovarian detection model named “SCBOD”. This model is divided into four stages, with the cleaning of the ultrasound visual data serving as the first stage. In the second stage, the segmentation technique is used for object detection and selection. In the third stage, based on the architectural and analytical properties of the object, SCBOD performs accurate recognition by utilizing a variety of metrics such as dimension, weight, average, variance, etc. In the end, an SVM is used for classification to conclude either PCOS or non-PCOS. Moreover, the authors in [36] used multi-level thresholding to extract geometrical features from ultrasound images. Then, the extracted features are passed to a supervised learning algorithm to classify an image as PCOS or non-PCOS. Recently, a multi-stack machine learning model along with explainable artificial intelligence [37] techniques such as QLattices, ELI5, LIME, etc., has been utilized for the recognition of PCOS. In [38], an ensemble-based framework using five traditional machine learning models for training and testing while obtaining the most dominant features, involving several approaches such as PCA and Chi-Square, was employed to obtain the most accurate prediction from a dataset. The method in [39] also introduced machine learning-based techniques, but the prediction of PCOS was conducted with the help of tongue and pulse readings. Conversely, the authors of another study identified PCOS with a variety of symptoms such as hypertension, diabetes, and other cardiovascular diseases using machine learning [40]. In this study, researchers combined two existing public datasets to generate a new dataset. The disease has been identified for the eight features chosen, using both supervised and unsupervised methods, after feature selection.

It is a matter of fact that convolutional neural networks (CNNs) perform better on visual data [41,42,43]. Therefore, the IAKmeans-RSA approach investigated in [44] has been proposed for use in the segmentation of cysts based on visual inputs and the identification of follicles. A CNN was used for acquiring all of the relevant aspects from the pictures that were segmented. In the final step, the categorization is carried out via an approach known as a deep neural network (DNN). In addition to extensive research based on textual data like pulse readings, a great deal of work has been conducted based on visual examples. Ultrasound pictures have been used in [45] to make predictions on PCOS using hybrid deep learning-based models. An ensemble deep learning-based model has been applied on ultrasound images for PCOS prediction. This work introduced a hybrid CNN that utilized pre-trained ResNet-50 and VGG-16 parameters to estimate the likelihood of PCOS. Both the pretrained VGG-16 and ResNet-50 architectures developed by receiving an input that comprised an MRI picture. After that, an outcome of the last max pooling layer in the VGG-16 model was transmitted to a fully connected (FC) layer that was equipped with an ReLu activation function. Simultaneously, the result of the final average pooling layer of ResNet-50 is fed into the FC layer that was equipped with an ReLu. A summary of some of the selected state-of-the-art related works is presented in Table 2. 

### 2.1. Motivation

As the literature reveals, the bulk of the research effort in this subject is carried out using machine learning techniques. Gaining the motivation from this literature review, we have proposed automated PCOS prediction models that are based on SMOTE for dataset balancing, a 1D CNN and LSTM. In our research, PCOS clinical data available via the Kaggle repository are used. The detection and prediction of PCOS are the primary concerns of our study. It is crucial to acknowledge that PCOS can change lives and may lead people to suffer for a very long time. It is essential to identify it more accurately and precisely and we need better solutions for a better and early diagnosis. The usage of machine learning algorithms is the main focus of the majority of existing research related to PCOS. It is worth noting that handcrafted feature engineering makes it difficult for machine learning algorithms to produce reliable categorization with high precision and accuracy. Therefore, there is need to have an efficient model that can deliver automatic feature extraction, is lightweight, and offers great precision in comparison to the most cutting-edge approaches to tackling PCOS problems. This study uses lightweight models for feature extraction, which is considered the toughest step in any machine learning algorithm; that is why more focus is given to this step only. The other challenges include (i) the imbalanced nature of the dataset and (ii) not properly scaled and normalized data.

The objective of this work is to use a lightweight deep learning model and compare these models to find out and select the best one among them. Other strong models like BERTs, XlNet, etc., require heavy processing, which is not feasible for such a small dataset.

### 2.2. Our Contributions

Here, a novel and lightweight deep learning model is suggested for automated PCOS prediction after reviewing the related research studies that exist in the literature. In comparison to almost all other machine learning-based PCOS detection and detection solutions available so far, the proposed model outperforms them in terms of performance, is simple, and has a small number of trainable parameters. The main contributions of this work are as follows:

- Multiple approaches are used to preprocess a highly unbalanced dataset in order to make it suitable for high-performance deep learning models.
- Instead of manually built methodologies, automatic feature extraction models based on LSTM and a customized 1D CNN are proposed.
- To enhance PCOS prediction accuracy, three separate and simple yet effective deep learning models are built, applied, and analyzed.
- To highlight the superiority of the proposed DL model, several existing PCOS detection methods are contrasted with this rendition.

## 3. Materials and Methods

In this section, the various materials and tools used to cultivate the proposed PCOS prediction models are described. In the proposed models, the CNN, LSTM, and SMOTE are predominantly engaged.

### 3.1. Convolutional Neural Networks

A one-dimensional CNN is a type of deep learning model that is used for processing sequences of data, such as time-series data or sequences of words in natural language processing. Unlike traditional 2D CNNs that are designed for image processing, 1D CNNs are designed to operate on sequences of data and have a convolutional operation that is performed along the time dimension. A 1D CNN consists of multiple layers, including an input layer, hidden layers with 1D convolutional and pooling operations, and an output layer. The convolutional operation computes the dot product between a filter and input sequence segment by sliding it along the time axis. This procedure extracts the local correlations in the input sequence, which helps the CNN to learn data patterns. Conversely, pooling reduces the input sequence resolution, compacting the representation and hence decreasing the computing cost incurred. Moreover, 1D convolutional neural networks are widely utilized to process audio, speech, natural language, and time-series forecasting for different areas of applications. Their architecture enables the developer to adjust the layer count, filter size, activation functions, etc.

### 3.2. LSTM

LSTM is a lightweight deep learning network, which has a sort of recurrent neural network (RNN) architecture. LSTMs are considered ideal for sequence-based data and long-term storage. Instead of losing information as input sequences lengthen, LSTMs possess memory cells that can hold information for a long duration and prevent the problem of a vanishing gradient. LSTMs have been widely incorporated for speech recognition, language translation, and sentiment analysis, etc. They are also very helpful in handling time series data and have the ability to capture sequence dependencies very well.

### 3.3. SMOTE

SMOTE is a powerful machine learning method intended for imbalanced datasets [43]. SMOTE stands for Synthetic Minority Over-Sampling Technique. SMOTE can be understood as an advanced version of over-sampling or data augmentation. It generates synthetic data points along with the original data points. The major advantage of SMOTE is that the synthetic data points generated are not duplicate values of the original ones but are slightly different from the actual data points. This helps to balance class distribution, which can eventually lead to an improved performance of the model especially for those datasets that suffer from imbalance. The dataset considered for our research has a slight imbalance, with less data in the infected class than in the not-infected class. Hence, SMOTE proves to be highly beneficial. SMOTE is advantageous in preventing over-fitting as well alleviating the issue where ML models may find it difficult to learn patterns due to the scarcity of data in a particular class. The SMOTE algorithm randomly selects a sample from the minority class. Secondly, k-nearest neighbors are found for every observation in the minority class data. A vector is identified between the actual data point and the neighbor. The next step is to multiply the vector with a random value between 1 and 0. Finally, to generate a synthetic data point, the value after multiplying the vector to a random value between 0 and 1 is added to the current data point.

### 3.4. DeLong Test

The DeLong test is a statistical method for comparing the performance of two correlated ROC (receiver operating characteristic) curves. It assesses whether the difference between the areas under the curve (AUCs) for the two models is statistically significant. The AUC is a measure of how well a classification model performs, with larger values indicating stronger performance. The DeLong test provides a *p*-value, which helps determine if the AUC difference is meaningful. If the *p*-value is below a certain threshold (like 0.05), it suggests a significant difference between the models. A higher *p*-value implies that the difference may be due to random variation.

## 4. Proposed Methodology

The suggested method for PCOS prediction will be explained in this section. A brief summary of the dataset is presented in this section, which is followed by a detailed description of the suggested deep learning-based models.

### 4.1. Dataset Description

The collection of polycystic ovary syndrome (PCOS) diagnosis data used in this paper is provided by Kottarathil and is available via the Kaggle repository [50]. The dataset contains diagnostic and thorough information on 541 individuals. The work mainly focuses on screening and diagnosing, and it includes a file that contains physical and clinical factors related to PCOS. The data contain 45 factors, out of which two of them have been recognized as unique identifier values and one factor contains NULL values; thus, they have been removed. The target variable “PCOS” is binary as positive cases are represented by 1 and negative cases are represented by 0. An inconsistent record for a patient and other non-numerical information were removed from the dataset during cleaning, leaving a final count of 540 samples. We use SMOTE to resample our data for maintaining a balanced distribution of losses in the testing and training dataset. The dataset is split in such a way that 80% of the data are used for training and 20% used for testing.

### 4.2. Preprocessing

**Figure 1:**
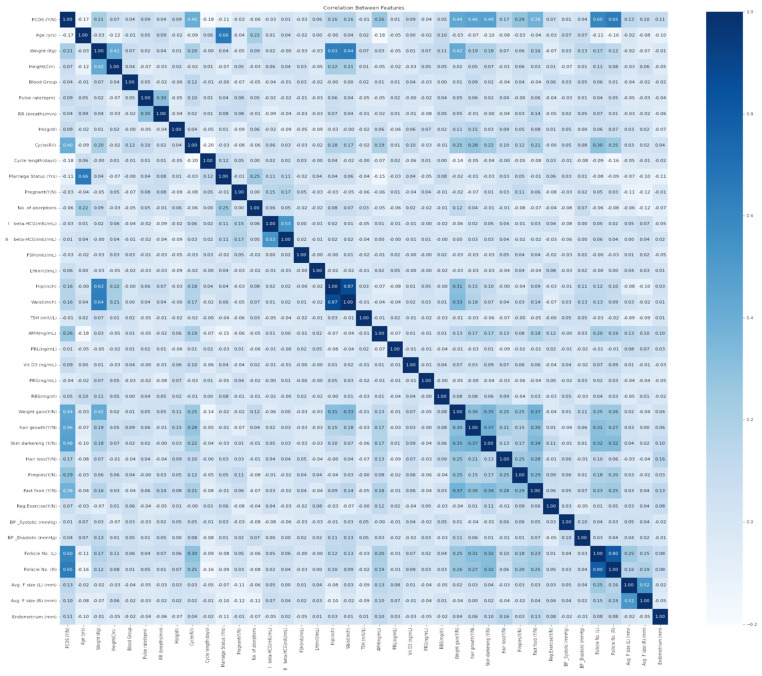
Heatmap for examining the correlations between all features of the PCOS dataset.

**Figure 2:**
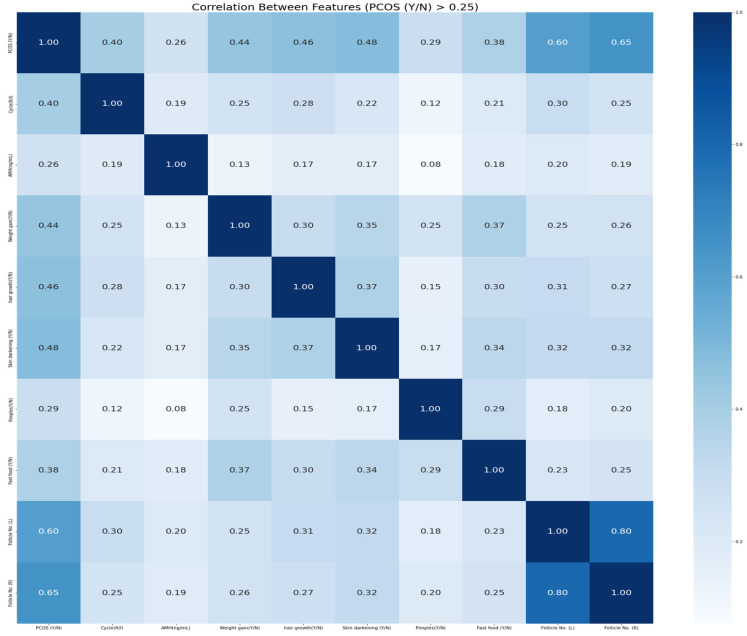
Heatmap for the correlations between the features with threshold 0.25 of PCOS dataset.

In order to improve the quality and relevance of the data, a correlation analysis is achieved on the PCOS diagnosis dataset. This analysis aims to find columns with poor relationships with other variables of the dataset. Accordingly, a correlation analysis is executed using the Pearson correlation coefficient among different variables of the dataset. The heatmap, showing the underlying correlations, is shown in Figure 1. Columns having a correlation value below 0.1 were eliminated since they had little effect on the data. Hence, all columns with a correlation equal to or higher than 0.1 and up to 1.0 are taken into consideration. After removing such columns, the dataset was found to have 35 columns. This is a widely used procedure in feature selection. Notably, removing features with a low association with the target variable or other attributes streamlines the dataset for accurate modeling. The correlation value of 0.1 is conservatively selected to exclude only features with a weak association. This reduces the dataset and may make PCOS diagnosis without infertility more predictive. An example correlation analysis with a threshold of 0.25 is depicted through the heatmap shown in Figure 2.

Data preprocessing also involves detecting and deleting percentile outliers so as to better the modeling accuracy and stability. A boxplot is used to reduce outliers in the “beta-HCG (mIU/mL)” feature. Identifying and managing outliers can improve statistical analysis and modeling accuracy. The boxplot approach depicts the median, quartiles, and outliers as points outside the quartiles. Outliers are observations with “beta-HCG (mIU/mL)” values below the 0.85 percentile. These data are dropped to decrease the impact of extreme scores on the analysis. As a result, the dataset has 458 rows instead of 541 after removing such outliers.

To resolve the class imbalance in the PCOS dataset, the Synthetic Minority Over-Sampling Technique (SMOTE) is adopted for the purpose. To maintain uniform class distribution, SMOTE creates synthetic samples of the class with a low number of samples (class 1). Our dataset contains 314 samples of class 0 and 144 of class 1. In general, the learning models may be biased toward the class with a higher number of samples and perform poorly on the other class due to the imbalance. SMOTE synthesizes class 1 samples by interpolating their feature values with a randomly selected same-class sample. Artificial samples are introduced into the dataset to boost the minority class samples. Eventually, SMOTE balances class 0 and class 1 with 314 samples each. The 628-row dataset has a better and uniform class distribution, which helps the learning models to perform in an unbiased manner.

Machine learning models are very sensitive to the input variable scale, which can affect their performance. Therefore, data normalization is carried out using the standard scaling method. Standard scaling assigns a variable a zero mean value and 1 as the standard deviation. To do this, it removes each feature’s mean from its values and divides it by its standard deviation. To avoid affecting analysis or modeling, it ensures that each feature has a similar scale. Each feature is changed to have a mean of zero and a standard deviation of one after standard scaling to ensure fair treatment in the analysis and modeling. This preprocessing step is considered to have better resilience and accuracy.

Next, the PCOS dataset is split 80:20 across training and testing sets. Most learning models divide data into training and testing sets to evaluate model performance on unknown data. The data were divided into a training set comprising 270 instances of class 0 and 100 instances of class 1 and a test set with an equal distribution of 44 instances for each class.

This balanced split ensured a proportional representation of the classes for both model training and evaluation purposes. To resolve the class imbalance in the PCOS training dataset, the Synthetic Minority Over-sampling Technique (SMOTE) is adopted for this purpose. To maintain a uniform class distribution, SMOTE creates synthetic samples of the class with a low number of samples (class 1). Our train dataset contains 270 samples of class 0 and 100 of class 1. In general, the learning models may be biased toward the class with a higher number of samples and perform poorly on the other class due to the imbalance. SMOTE synthesizes class 1 samples by interpolating their feature values with a randomly selected same-class sample. Artificial samples are introduced into the dataset to boost the minority class samples. Eventually, SMOTE balances class 0 and class 1 with 270 samples each. The 540-row dataset has a better and uniform class distribution, which helps the learning models to perform in an unbiased manner. Next, transforming the training set and label prepared the PCOS diagnosis dataset for the learning model. The shape of the input features of train and test datasets is indeed transformed to (540, 35, 1) and (88, 35, 1), respectively. This three-dimensional structure reflects the incorporation of an additional dimension, specifically introduced to accommodate the sequential or time-series nature of the data. Each data instance is now represented as a 2D matrix, where rows correspond to different time steps, columns represent distinct features, and the third dimension signifies the presence of a single channel. This reshaped format aligns with the input requirements of certain machine learning models, particularly those designed to handle sequential data, such as recurrent neural networks (RNNs) or convolutional neural networks (CNNs) for time-series analysis. The resulting shape (540, 35, 1) and (88, 35, 1) will be crucial for subsequent model training and evaluation. This transformed representation of the label allows for the use of supervised learning algorithms for binary classification, where the goal is to predict one of two classes. By transforming the shape of the training set and label, the PCOS diagnosis dataset is prepared for input into a deep learning model, which can then use the transformed data to learn patterns and make predictions.

### 4.3. Proposed Models

**Figure 3:**
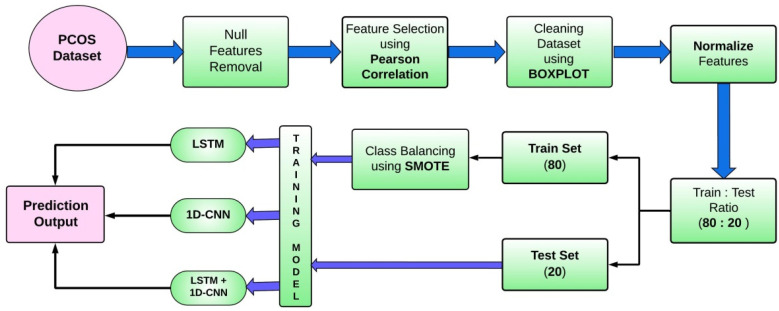
Schematic diagram of the proposed methodology.

**Figure 4:**
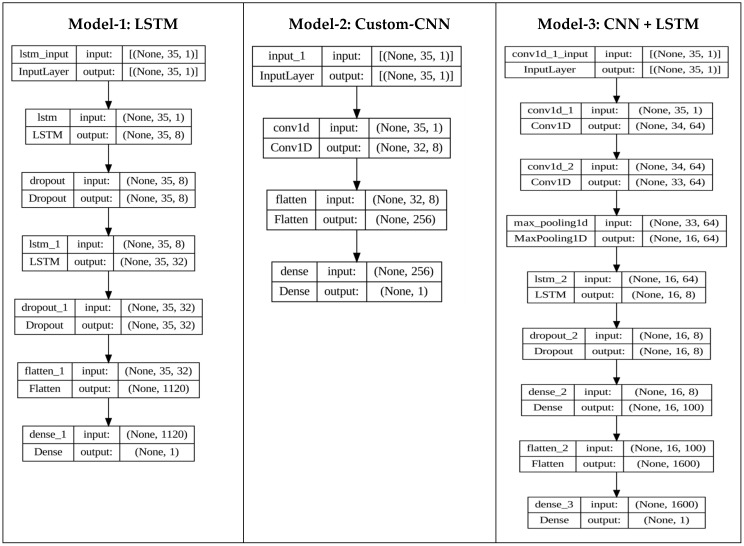
Typical architectures of the three proposed deep learning models with the size of input and output of each layer.

To have a better medical approach for the diagnosis of PCOS, three lightweight deep learning models are analyzed to assess their performance and suitability for an accurate PCOS prediction and classification. A block diagram of the proposed methodology is shown in Figure 3. Moreover, architectural diagrams of all three models are shown in Figure 4. We began with a conventional LSTM as our first model and used SMOTE alongside it. It is a six-layer network. The second model is a custom CNN and the third one is a combination of the CNN and LSTM, respectively. Model-2 has one convolution layer while the third model has three convolutions and one LSTM layer. The reason behind using the three models individually is to even out any bias towards any model. In the proposed model-2, we custom-built a 1D convolutional neural network comprising four layers for an efficient PCOS classification These four layers include Conv1D, Dense and Flatten layers. The purpose of these layers is explained as follows: It begins with an input layer designed to accommodate input shapes of (35, 1), effectively capturing the temporal nature of the data. The Conv1D layer follows, featuring eight filters, a kernel size of 4, and a tanh activation function. This layer plays a key role in extracting local patterns from the sequential input and produces an output shape of (32, 8). The subsequent Flattening layer then reshapes this output into a one-dimensional tensor with dimensions of 256, optimizing the data for further processing. The last layer functions as a Dense layer, serving as the output for binary classification with one neuron and utilizing a sigmoid activation function. This simplified structure, containing only 297 trainable parameters and lacking non-trainable elements, prioritizes computational efficiency while effectively capturing important local patterns essential for analyzing sequential data. Therefore, the model provides an intentional and resource-efficient approach for extracting significant insights from a sequential data network. The time complexity of the CNN architecture was assessed using factors such as the trainable parameters (T), batch size (B), and epochs (E). If we assume the time complexity for a single forward and backward pass is O (T), then the overall training time complexity can be estimated as O (T × B × E). This led to effective training, with a total duration of 11.0 s.

Pseudo Code:

Step 1: Gather data.

Step 2: Preprocess data:

- Null removal.
- Feature selection (Pearson correlation).
- Cleaning (box plot).
- Normalization (standard normalization).

Step 3: Split data into the train and test set.

Step 4: Upsample the train set (SMOTE upsampling).

Step 5: Train all three models on train set.

- Train the LSTM model.
- Train the CNN model.
- Train the stacked LSTM, CNN model.

Step 6: Evaluate the model using the test set.

Step 7: Select the best model based on the test set and save the same one for later uses.

## 5. Performance Analysis

This section presents the findings of the experiments conducted and explains the various parameters like accuracy, precision, recall, etc. The evaluation metrics employed to measure the performance of the suggested model encompass *accuracy, recall, precision*, F1-score, and *AUC*, which are defined as follows:

Accuracy is articulated as follows:

$$\mathrm{ACC}=\frac{\mathrm{TP}+\mathrm{TN}}{\mathrm{TP}+\mathrm{TN}+\mathrm{FP}+\mathrm{FN}}$$

Recall is a parameter that gives the number of true positives out of all the positives obtained. Recall provides insights into the algorithm’s ability to detect relevant information and is also acknowledged as true positive rate or sensitivity.

$$\mathrm{Recall}=\frac{\mathrm{TP}}{\mathrm{TP}+\mathrm{FN}}$$

Precision refers to the ratio of true positives to all positives.

$$\mathrm{Precision}=\frac{\mathrm{TP}}{\mathrm{TP}+\mathrm{FP}}$$

F1-score is another significant performance indicator, which is the harmonic mean of precision and recall.

$$F1\text{-}\mathrm{score}=\frac{2\times \mathrm{Precision}\times \mathrm{Recall}}{\mathrm{Precision}+\mathrm{Recall}}$$

The area under the curve (AUC) indicates the level of separation or discriminability. It shows the effectiveness of our model in telling apart the different classes. A higher AUC value signifies better classification performance, accurately distinguishing between healthy and patient classes.

### 5.1. Simulation Results

The test was conducted using various tuning parameters, and the results were taken for each setting. The optimal parameters obtained for the fine-tuning of the models were taken as the final setting and were as follows: epochs were set to 50, the learning rate was set to 0.01, the optimizer used was Adam, the loss function used was binary cross entropy, gradient descent was applied on batch of 32, etc. These are listed in Table 3.

**Figure 5:**
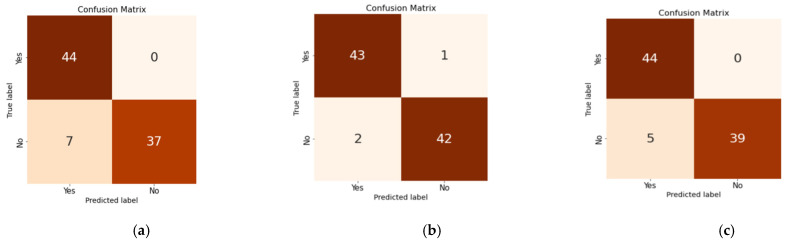
Confusion matrices for the proposed models. (**a**) LSTM, (**b**) Custom CNN, (**c**) CNN + LSTM.

The performance results obtained for the given setting are recorded and presented in Table 4. The table shows tests for various parameters like accuracy, precision, recall, F1-score and the AUC. An analysis of the parameters with the help of plots and confusion matrices is also presented. The confusion matrices of each model are shown separately, which gives the idea of the true positives identified. Plots of accuracy and loss against the number of epochs are also plotted, for both the train and test data. The confusion matrices for all three suggested models are shown in Figure 5. The accuracy measures obtained for the proposed three models for PCOS prediction are 92.04%, 96.59%, and 94.31%, respectively. In our model, recall represents the proportion of correctly identified patients out of all of the patients obtained. The peak recall achieved by model-1 is 92.04%, for model-2, it is 96.59%, and for model-3, it is 94.31%, respectively. Precision represents the proportion of accurately identified patients with PCOS out of all of the individuals identified as patients. The precision values for the three models are 93.13%, 96.60%, and 94.89%, respectively. Precision provides a count of the relevant data points and aids in evaluating the model’s accuracy. Figure 5 shows that model SMOTE + CNN gives the best results of all. In addition, it is evident from the confusion matrix that the precision, recall, F1-score, and AUC also show the best scores for this model. The stack of SMOTE and CNN performs best for all the given parameters. A one-dimensional CNN is used in order to extract features. Model-3 (SMOTE + CNN + LSTM) also performs well but slightly less well than the previous stack. The model SMOTE + LSTM shows the least convergence of all three. The reason behind the CNN performing better than the other two models is that CNNs are naturally spatial feature-extracting bodies. Conversely, LSTM is capable of catching temporal dependencies. The stack CNN + LSTM shows good results but inserts temporal dependency on top of spatial dependency. This adds another level of complexity on top of the CNN. Thus, the CNN outperforms the CNN + LSTM stack.

**Figure 6:**
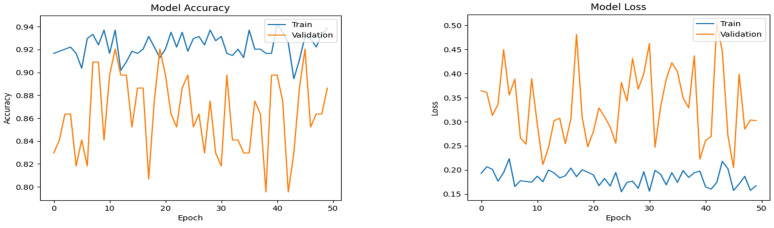
Accuracy and loss plots for the proposed LSTM model.

**Figure 7:**
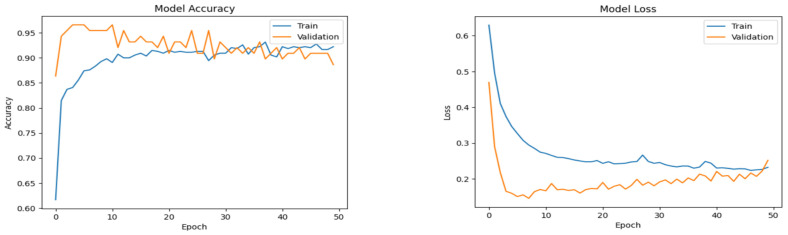
Accuracy and loss plots for the proposed custom CNN model.

**Figure 8:**
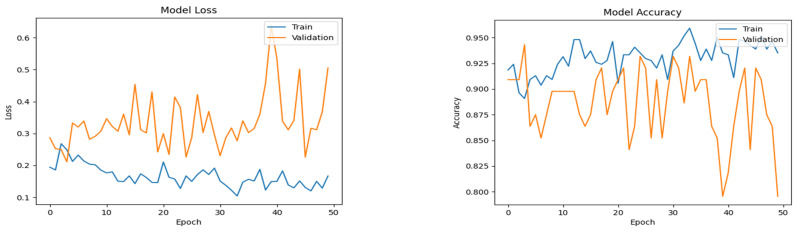
Accuracy and loss plots for the proposed custom CNN+LSTM model.

**Figure 9:**
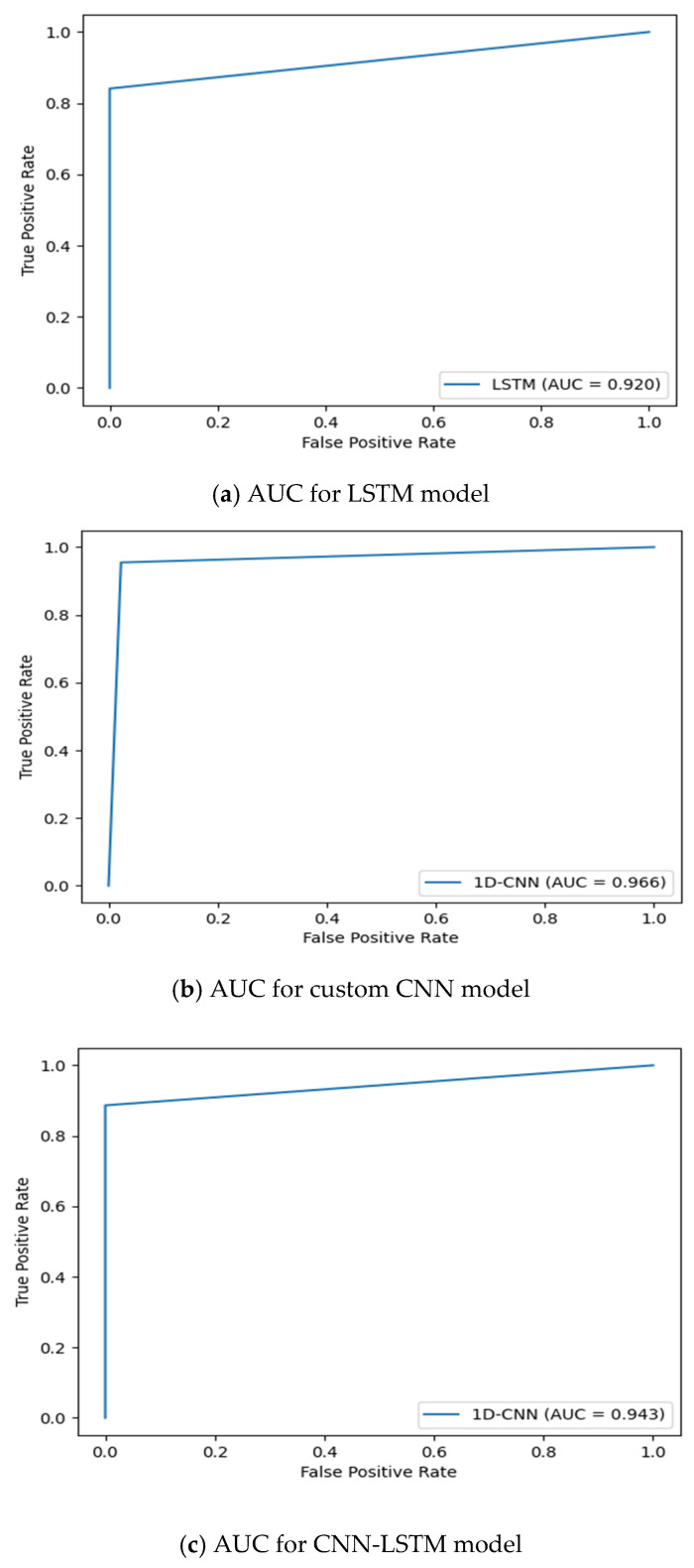
ROC-AUCs for PCOS prediction using (**a**) LSTM model, (**b**) custom CNN model, and (**c**) CNN-LSTM model.

The plots in Figure 6, Figure 7 and Figure 8 show the progressive convergence of the models with respect to epochs. The plots of accuracy vs. epochs show the best result for the SMOTE + CNN model; the model has low variance. The training and testing curves for this model have a smaller gap between them. The other models show noticeable variance between the training and testing data. The best accuracy of 96.59% was achieved using the SMOTE + CNN model and the other two models showed a slight lower side in the accuracy score. The behavior of loss incurred vs. epochs is also shown in Figure 5, Figure 6 and Figure 7. The training loss for the SMOTE + LSTM model converges abruptly, whereas the testing loss shows little convergence. The SMOTE + CNN model gives the best convergence among all, with less variation and low bias. The test set loss does not vary from the training set loss in this model. The stack of SMOTE + CNN + LSTM also shows visible variance when subjected to the training and testing set. The AUC behavior of the anticipated algorithm is depicted in Figure 9. The AUC obtained by model-1 is 92.0%, for model-2, it is 96.60%, and for model-3, it is 94.3%, respectively. The plots give an overview of the AUCs for all three models; all three models perform well regarding this objective. Again, the SMOTE + CNN model outperforms all the others, with a peak score of 96.59%. Thus, we can conclude that, given the results and after conducting various analyses, model-2, SMOTE + CNN, outperforms the other two models.

Our model is highly efficient, particularly in its lightweight design, which allows it to be trained using fewer parameters compared to those in previous studies. The CNN model trains with just 297 parameters, whereas the LSTM model uses 6689 parameters, and the combined CNN + LSTM model requires 13,285 parameters. In addition to its parameter efficiency, the proposed model is also time-efficient, completing its training in only 10.02 s, significantly faster than the LSTM model (67.27 s) and the CNN + LSTM combined model (18.51 s). Table 5 presents the efficiency metrics, including the computational resources utilized by each model, the number of trainable parameters, and the training duration. All three models exhibit a significant difference in their AUC values, as indicated by the *p*-values. The *p*-value for the comparison between the CNN and LSTM models is 0.038, between the CNN and the combined CNN + LSTM model is 0.043, and between the LSTM and combined CNN + LSTM model is 0.05. Table 6 provides a detailed breakdown of these *p*-values.

### 5.2. Comparison Analysis

**Figure 10:**
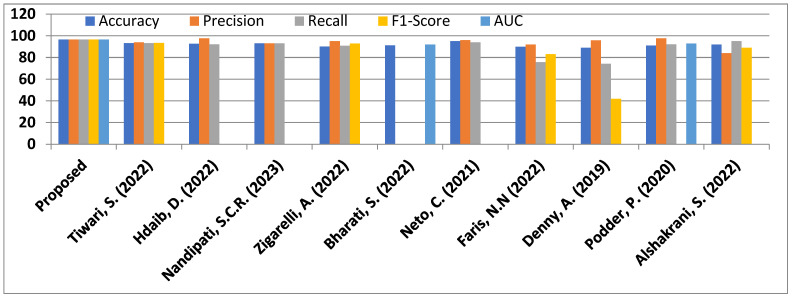
Performance comparison of PCOS prediction models [22,27,30,47,48,49,51,52,54].

A fair comparison of performance on the same dataset is necessary to judge the efficacy of the suggested model. This section aims to present a comparison analysis with some existing and recent PCOS detection models. Table 2 shows the PCOS detection ability of different models, which are obtained on the same Kaggle dataset [50] and performance is compared in terms of the standard performance metrics discussed earlier. Ref. [23] developed an SPOSDS, which is a smart diagnostic system for PCOS, by comparing the performance of many machine learning classifiers. The best accuracy of 93.25 has been presented by the SPOSDS system with the help of random forest (RF), using *sqrt* as the maximum feature hypermeter. Recently, Hdaib et al. in [27] also investigated different machine learning classifiers to have an effective solution for good PCOS detection. They were able to achieve an accuracy of 92.6% and precision of 97.6% using the linear discriminant analysis (LDA) technique. In Ref. [30], the authors analyzed a number of ML approaches to solve the accurate PCOS detection problem. Techniques such as KNN, SVM, RF, naïve Bayes, a neural network, bagging, and Adaboost were examined. The best performance (accuracy = 93.12%, precision = 93.12, and recall = 93.12%) were obtained for the random forest classifier with 40 features. Zigarelli et al. suggested a self-diagnostic model for PCOS in ref. [47], which provided an accuracy of 90.1% and precision of 95% among different scenarios of analyses. However, a different set of ensemble classifiers were studied such as voting hard, voting soft, and CatBoost in ref. [48]. The investigation and analysis found that the highest accuracy of 91.12% was achieved using voting soft for predicting patients with PCOS. Neto et al. in [51] examined various classifiers with the CRISP-DM model to predict PCOS. Their study reported the best classification performance through random forest and a data sampling technique, with an accuracy of 95%, precision of 96%, and recall of 94%. Recently, a genetic algorithm-based SVM method was suggested for PCOS classification in [52]. The performance results were not very convincing. Ref. [49] also reported a PCOS detection and prediction study using different machine learning classifiers. A low accuracy of 89.02% was achieved with a precision of 95.83%. In Ref. [53], the authors recommended a technique based on a hybrid of random forest and linear regression (RFLR), which was able to offer an accuracy of 91.01%, precision of 97.6%, recall of 92.2%, and area under the curve of 92.9%. In Ref. [54], a number of ensemble models such as HRFLR, extreme boosting with RF, linear SVM, light gradient boosting, and CatBoost were investigated for identifying PCOS. The analyses showed that CatBoost performed best among all of the ensemble models and provided an accuracy of 92% and recall of 95% but had a precision of 84% only. However, our proposed model-2, which is based on a lightweight 1D CNN, is able to predict PCOS in much better way as it offers an accuracy = 96.59%, precision = 96.60%, recall = 96.59%, F1-score = 96.59%, and AUC = 96.60%. A summary of the comparison study in terms of performance results such as accuracy, precision, recall, F1-score, the area under the curve (AUC) is presented in Table 7. The overall prediction performance of our proposed deep learning model is sufficiently more enhanced than all of the PCOS models listed in Table 7. The performance comparison is also graphically presented in Figure 10. Hence, the comparison analysis validates the better performance of the proposed model over many recent PCOS prediction and detection models.

### 5.3. Discussion

In our study, we used three models in order to make a bias invariant prediction. The model that gives the best results, i.e., (SMOTE + CNN), is selected as the final predictor. The dataset is split into train and test parts and SMOTE is applied on the train set, whereas test set is kept as it is. The data are also normalized and scaled because, as seen from the research, deep learning works very well with normalized and scaled data. The dataset goes through different preprocessing steps, like NULL feature removal, the Pearson correlation feature selection technique, etc. The model has a peak accuracy of 96% and strong convergence on both the test and train datasets. These diagrams additionally provide additional important results from the final model. The experiment demonstrated that the use of a CNN had a discernible impact on this dataset; the model that employed a CNN as a feature extractor demonstrated higher convergence. We tried to reduce the loss, but after a few epochs, it flattened out. For this reason, we prematurely ended our model (early stopping) to prevent overfitting. The final model is stored for future use.

## 6. Conclusions

Presently, a variety of machine learning classifiers and ensemble-based methods have been investigated for PCOS diagnosis and prediction. In practice, hand-crafted feature extraction through ML-based approaches had been exhaustively suggested, which have low-performance difficulties that can be disregarded for the precise diagnosis and prediction of PCOS. In order to detect PCOS more accurately, this paper proposed automated feature engineering based on lightweight deep learning models. For the better performance and excellence of the medical systems used for the diagnosis of PCOS, the proposed PCOS prediction method suggests three lightweight deep learning models based on LSTM and a customized 1D CNN for feature extraction instead of manual extraction via ML approaches, wherein different data preprocessing steps are performed on a highly unbalanced dataset in order to prepare it to have valid and high performance. In an effort to increase the accuracy of PCOS prediction, three different yet efficient lightweight deep learning models are designed and examined. Our specially designed 1D CNN-based model found to present the highest accuracy (96.59%), precision (96.60%), recall (96.59%), F1-score (96.59%), and ROC-AUC (96.60%), among all three proposed models. The proposed model is highly time-efficient, finishing its training in 10.02 s. Additionally, it utilizes the fewest parameters (297) compared to the other two models, further enhancing its efficiency, and the DeLong test results for comparing the AUCs of the anticipated models show its statistical significance and relevance. As a result, the suggested model offers the best performance, which may assist in identifying PCOS early. In addition, the proposed model possesses superior performance when compared with a number of recently created existing PCOS prediction and detection learning models.

## Figures and Tables

**Table 1:** List of abbreviations.

Different Abbreviations	
PCOS	Polycystic Ovarian Syndrome
SMOTE	Synthetic Minority Over-Sampling
CNN	Convolutional Neural Network
LSTM	Long Short-Term Memory
SCBOD	Size and Count-Based Ovarian Detection
KNN	k-Nearest Neighbors
SVM	Support Vector Machine
ReLu	Rectified Linear Unit
RNN	Recurrent Neural Network
AUC	Area Under the ROC Curve
ROC	Receiver Operating Characteristic Curve

**Table 2:** Summary of related works.

Author(s)	Methodology	ML Approach	Key Findings/Contributions
Ref. [1] Azamossadat Hosseini, 2023	Comparative analysis	Segmentation with K-means clustering deep CNN	The model achieved 100% accuracy, sensitivity, and specificity in classifying B-ALL cases, leading to the development of a mobile application for real-time screening.
Ref. [18] Mehdi Gheisari, 2024	Systematic review of studies using PRISMA protocol	AI-based diagnosis using deep learning techniques	The CNN outperformed other AI techniques in processing healthcare data, making mobile apps a crucial tool for early COVID-19 detection and future pandemic management with AI and advanced technologies.
Ref. [19] Shubham Bhosale, 2022	Noise reduction and segmentation techniques	Deep convolutional neural networks (DCNNs)	A DCNN was applied to improve cyst diagnosis accuracy by reducing noise, extracting the region of interest, and enabling the early detection of PCOS-related anomalies to prevent infertility.
Ref. [24] MM Hassan, 2020	Comparative analysis	Random forest algorithm	This involved the use of a random forest algorithm used in the diagnosis of PCOS, with an accuracy of 96%.
Ref. [25] Dana Hdaib, 2022	Comprehensive comparative analysis	K-nearest neighbors	The study presented a milestone for building a completed CAD system for the problem.
Ref. [31] Ning-Ning Xie, 2020	An integrated Machine learning methodology	Random forest and artificial neural network	The model demonstrated improved predictive accuracy in microarray data compared to the use of conventional marker genes.
Ref. [33] Zozan Guleken, 2022	Raman spectroscopy and multivariate analysis	Principal component analysis (PCA)	The findings indicated that the lipid and protein equilibrium could serve as a valuable indicator for PCOS in Raman spectra.
Ref. [34] Beny Cahyono, 2017	Automated analysis of ultrasound	Deep convolutional neural network	This gave a solution that incorporated automated feature extraction using a convolutional neural network.
Ref. [45] P. Chitra, 2023	Transfer learning with hybrid models	ResNet-50 and VGG-16	The study introduced a combined model approach to improve training and precision, resulting in a 93% accuracy on the test dataset for predicting PCOS.
Ref. [46] Elreedy, D. 2019	Theoretical Analysis	SMOTE augmentaion, K-nearest neighbors	SMOTE generates synthetic samples along K-nearest neighbors to effectively balance datasets. The study that factors like data dimension and the number of neighbors influence accuracy and classification boundaries.
Ref. [47] Angela Zigarelli, 2022	Principal component analysis	CatBoost classification	The prospective study suggested that the self-diagnostic prediction models for PCOS status could function as a convenient and easily accessible digital platform utilizing existing health metrics, benefiting both prospective patients and healthcare providers.
Ref. [48] S Bharati, 2022	A data-driven approach	Ensemble learning	This study considered the use of data-driven methods in diagnosing PCOS illness in women.
Ref. [49] Amsy Denny, 2019	Comprehensive comparative analysis	Random forest classifier	The model achieved 89.02% accuracy in PCOS diagnosis using a RFC.

**Table 3:** Optimal parameters for fine-tuning of model.

Hyperparameters for Proposed Model	
Filters	8
Kernel Size	4
Activation Function	Tanh, Sigmoid
Loss Function	Binary cross-entropy
Learning Rate	0.01
Optimizer	Adam
Epochs	50
Batch Size	32
Number of Neurons in Dense Layer	1

**Table 4:** Performance of proposed models for PCOS classifications.

Proposed Models		Accuracy	Precision	Recall	F1-Score	AUC
Model-1	SMOTE + LSTM	92.04%	93.13%	92.04%	91.99%	92.0%
Model-2	SMOTE + CNN	96.59%	96.60%	96.59%	96.59%	96.60%
Model-3	SMOTE + CNN+ LSTM	94.31%	94.89%	94.31%	94.30%	94.3%

**Table 5:** Computational resources utilized by models and the training duration.

Model	Parameters	RAM (GB)	GPU (GB)	Time (Seconds)
SMOTE + LSTM	6689	12.7	15	67.27
SMOTE + CNN	297	12.7	15	10.02
SMOTE+ CNN+ LSTM	13285	12.7	15	18.51

**Table 6:** DeLong test for comparing AUC of PCOS model.

Combinations	Model	Model	*p*-Value
1	SMOTE + LSTM	SMOTE+ CNN+ LSTM	0.05
2	SMOTE + CNN	SMOTE + LSTM	0.038
3	SMOTE+ CNN+ LSTM	SMOTE + CNN	0.043

**Table 7:** Performance comparison of proposed model on same Kaggle dataset.

Model	Accuracy	Precision	Recall	F1-Score	AUC
Proposed	96.59	96.60	96.59	96.59	96.60
Ref. [23]	93.25	94.0	93.25	93.42	-
Ref. [27]	92.6	97.6	92.2	-	-
Ref. [30]	93.12	93.12	93.12	-	-
Ref. [47]	90.1	95.0	90.9	92.8	-
Ref. [48]	91.12	-	-	-	92.0
Ref. [51]	95	96	94	-	-
Ref. [52]	90	92	75.7	83	-
Ref. [49]	89.02	95.83	74.19	41.82	-
Ref. [53]	91.01	97.6	92.2	-	92.9
Ref. [54]	92.0	84.0	95.0	89.0	-

## Data Availability

The dataset of polycystic ovary syndrome (PCOS) diagnosis used in this paper is available via the Kaggle repository [50].
